# Beyond MRI: Unveiling metabolic signatures of primary hypophysitis with ^18^F-FDG PET

**DOI:** 10.1007/s40618-026-02907-2

**Published:** 2026-05-22

**Authors:** Serdar Sahin, Betul Banu Kocaman, Mehmet Ufuk Yanmaz, Said Erkam Biyikoglu, Ozge Polat Korkmaz, Emre Durcan, Sertac Asa, Haluk Burcak Sayman, Hande Mefkure Ozkaya, Necmettin Tanriover, Pinar Kadioglu

**Affiliations:** 1 Division of Endocrinology, Metabolism and Diabetes, Department of Internal Medicine, Cerrahpasa Faculty of Medicine, Istanbul University- Cerrahpasa, Kocamustafapasa Street No:53 34098, Fatih, Istanbul, Turkey; 2 Cerrahpaşa Faculty of Medicine, Department of Nuclear Medicine, Istanbul University-Cerrahpaşa, İstanbul, Turkey; 3 Department of Neurosurgery, Cerrahpasa School of Medicine, Istanbul University-Cerrahpasa, Istanbul, Turkey; 4 Pituitary Tumor Center of Excellence (PTCOE), Cerrahpasa Faculty of Medicine, Istanbul University-Cerrahpasa, Istanbul, Turkey

**Keywords:** Primary hypophysitis, ^18^F-FDG PET, SUVmax, SUVmean

## Abstract

**Aims:**

Hypophysitis is a heterogeneous clinical entity originating from different anatomical compartments of the pituitary gland and driven by diverse pathophysiological mechanisms. This study aims to explore the potential diagnostic and clinical value of ^18^F-fluorodeoxyglucose positron emission tomography in the evaluation of primary hypophysitis.

**Materials and methods:**

This single-center retrospective study was conducted at the pituitary referral unit of a tertiary university hospital. The study population included patients with primary hypophysitis, individuals with nonfunctioning pituitary adenomas, and subjects without intracranial pathology. Demographic features, baseline clinical and biochemical characteristics, as well as initial cranial and sellar magnetic resonance imaging and ^18^F-fluorodeoxyglucose positron emission tomography findings, were systematically analyzed and compared across groups. Furthermore, in the primary hypophysitis subgroup, potential associations between positron emission tomography imaging parameters and clinical, radiological, and laboratory characteristics were specifically examined.

**Results:**

A total of 46 participants were enrolled in the study, comprising 16 patients with primary hypophysitis, nine with nonfunctioning pituitary adenoma, and 21 controls without intracranial pathology. Positron emission tomography demonstrated significantly higher maximum and mean standardized uptake values in patients with primary hypophysitis compared with controls, whereas no significant differences were observed between the other groups. Within the primary hypophysitis cohort, mean standardized uptake values were significantly elevated in patients with hypopituitarism and in those with Arginine vasopressin deficiency (central diabetes insipidus) compared to their respective counterparts without these conditions. Furthermore, both maximum and mean standardized uptake values were markedly higher in patients with growth hormone deficiency than in those with preserved growth hormone function.

**Conclusion:**

These findings suggest that metabolic activity observed on ^18^F-fluorodeoxyglucose positron emission tomography may serve as a marker of disease severity and pituitary dysfunction in primary hypophysitis.

## Introduction

Hypophysitis is an uncommon inflammatory disorder involving the sellar and suprasellar regions, resulting in varying degrees of anterior and/or posterior pituitary hormone deficiencies [1]. In recent years, advances in diagnostic techniques have markedly improved the recognition and characterization of this condition. While initially considered a primarily autoimmune disease, hypophysitis is now understood as a heterogeneous entity with diverse etiologies, including autoimmune, inflammatory, infectious, and neoplastic causes, often with an unknown pathogenesis [2]. Its true annual incidence remains uncertain, with earlier estimates suggesting approximately 1 case per 9 million individuals [2]. The recognition of novel etiologies, the increased use and refinement of imaging modalities, and the rising number of pathological specimens obtained following pituitary surgery have all contributed to an apparent increase in reported cases [3, 4]. Accurate diagnosis can be challenging, as hypophysitis may present with clinical and radiological features that overlap with other pituitary lesions, such as common pituitary adenomas or rare pituitary metastases.

Positron emission tomography (PET) is an imaging modality increasingly used for diagnosis, staging, treatment planning, and monitoring of disease progression, particularly in oncology patients [5]. Beyond its oncological applications, PET can aid in the differential diagnosis of certain non-malignant conditions. Under normal circumstances, ^18^F-fluorodeoxyglucose (^18^F-FDG) uptake in the pituitary gland is minimal or absent [6], likely due to the gland’s small size and relatively low metabolic activity. However, certain benign and malignant pituitary lesions may demonstrate increased ^18^F-FDG uptake, many of which are detected incidentally during cancer imaging studies [7–11]. Although ^18^F-FDG PET imaging can provide valuable information in the evaluation of hypophysitis, most studies to date have focused on immunotherapy-induced hypophysitis. Data specifically addressing the role of ^18^F-FDG PET in primary hypophysitis remain scarce. Therefore, the present study aimed to investigate the potential utility of ^18^F-FDG PET in the evaluation of primary hypophysitis.

## Materials and methods

This single-center retrospective study was conducted at the pituitary center of a tertiary care university hospital and included patients evaluated between 2007 and 2024. The study was approved by the Local Ethics Committee of Cerrahpasa Faculty of Medicine and conducted in accordance with the principles of the Declaration of Helsinki.

### Participants

The study population consisted of three groups: patients with primary hypophysitis, patients with non-functioning pituitary adenomas (NFPAs), and control subjects without intracranial pathology. Eligible participants were adults between 18 and 65 years of age. Patients with primary hypophysitis who presented to our clinic were retrospectively evaluated based on medical records. Patients with a diagnosis of primary hypophysitis established on the basis of clinical, radiological, and/or histopathological criteria were included in the study. In these patients, ¹⁸F-FDG PET was performed as part of the diagnostic workup to exclude secondary causes of hypophysitis, including systemic inflammatory, granulomatous, or malignant diseases, and to assist in differentiating primary from secondary hypophysitis. Exclusion criteria included the absence of ^18^F-FDG PET imaging, the presence of secondary hypophysitis, initiation of treatment prior to ^18^F-FDG PET imaging, or insufficient FDG administration—such as cases involving extravasation or inadequate tracer activity. Patients with NFPA were identified according to current guidelines [12, 13] and were included only if ^18^F-FDG PET imaging had been performed prior to any intervention. These patients underwent ^18^F-FDG PET imaging due to systemic symptoms such as weight loss or constitutional complaints prompting investigation for an underlying malignancy. There was no clinical or radiological suspicion of sellar or parasellar metastasis. In some patients, the pituitary lesion was incidentally detected during imaging, whereas in others, a previously known NFPA was present. Functional adenomas, prior treatment, lack of ^18^F-FDG PET imaging or insufficient FDG administration—such as cases involving extravasation or inadequate tracer activity were grounds for exclusion. The control group comprised individuals who underwent ^18^F-FDG PET imaging for systemic symptoms such as anemia, weight loss, or night sweats, but in whom no underlying pathology was found, and who had no pituitary or other intracranial lesions on magnetic resonance imaging (MRI) or ^18^F-FDG PET imaging. Participants without cranial or sellar MRI, without ^18^F-FDG PET imaging, with any intracranial pathology, or with insufficient FDG administration (e.g., extravasation or inadequate tracer activity) were excluded.

Demographic characteristics, baseline clinical data, initial laboratory parameters, and findings from the initial cranial and/or sellar MRI and ^18^F-FDG PET imaging of participitans were analyzed and compared to each other.

Pituitary dysfunction was defined according to the current guidelines [14, 15]. Clinical examination, imaging, and laboratory investigations were performed to differentiate primary hypophysitis from secondary hypophysitis. Computed tomography (CT) of the chest, abdomen, pelvis and ^18^F-FDG PET/CT or ^18^F-FDG PET/MRI were used for the evaluation of malignancy. Serum angiotensin-converting enzyme (ACE) measurement and chest CT were utilized for the diagnosis of sarcoidosis, whereas the tuberculin purified protein derivative test together with chest CT was employed for the diagnosis of tuberculosis. Other autoimmune disorders were systematically investigated in patients with clinical suspicion; serum autoantibodies—including antithyroid antibodies (TPOAb, TGAb, TRAb), anti-transglutaminase/anti-endomysial antibodies, adrenal autoantibodies (AAA), islet cell autoantibodies (ICA), antibodies to insulin (IAA), glutamic acid decarboxylase antibodies (GAD), antinuclear antibodies (ANA), and autoantibodies associated with Sjögren’s syndrome (SSA-Ab, SSB-Ab) were measured.

Glucocorticoid therapy was recommended primarily for younger patients, those with partial or complete hypopituitarism, Arginine vasopressin (AVP) deficiency (central diabetes insipidus), or evidence of ophthalmological or neurological involvement. Conversely, a conservative approach was reserved for patients without ophthalmological or neurological manifestations, for those with major contraindications to glucocorticoid therapy, or in cases where a multidisciplinary risk - benefit assessment indicated a high likelihood of adverse events. At our center, glucocorticoid therapy is initiated with methylprednisolone at a dose of 120 mg/day for two weeks, followed by a gradual weekly taper to 80 mg, 60 mg, 40 mg, and 20 mg. The overall duration of this regimen is approximately six weeks and is adjusted according to clinical response and tolerability.

### FDG PET imaging protocols and analysis

All participants underwent whole-body ^18^F-FDG PET/CT or ^18^F-FDG PET/MRI imaging, covering the area from the vertex to the mid-thighs, using dedicated scanners (PET/CT: Discovery 710, GE Healthcare, Milwaukee, USA; PET/MRI: Signa 3 Tesla, GE Healthcare, Milwaukee, USA). Among the participants, one patient with primary hypophysitis and one patient with NFPA underwent ^18^F-FDG PET/MRI, while all remaining participants, including the control group, underwent ^18^F-FDG PET/CT. Following intravenous administration of ^18^F-FDG (activity adjusted to body weight), patients rested for approximately 60 min prior to image acquisition. The acquisition protocol included 2 min/bed position, using a VUE Point FX reconstruction algorithm with two iterations, eight subsets, and a 5.5 mm sharp IR filter. For quantitative analyses, volumetric delineation of the pituitary gland was performed on the non-contrast CT component of the ^18^F-FDG PET/CT images to ensure accurate anatomical localization. A volume of interest (VOI) was manually drawn to encompass the entire anatomical boundaries of the gland and subsequently projected onto the corresponding ^18^F-FDG PET images. From these VOIs, ^18^F-FDG PET-derived standardized uptake values were calculated, with SUVmax defined as the maximum voxel value within the VOI and SUVmean defined as the mean uptake activity across the VOI. The VOI volume itself was not systematically recorded.

### Statistical analysis

Statistical analyses were performed using the Statistical Package for the Social Sciences (SPSS), version 22.0. The normality of data distribution was assessed with the Kolmogorov–Smirnov test. Continuous variables with a normal distribution are presented as mean ± standard deviation (sd), whereas those without a normal distribution are reported as median [interquartile range, IQR]. Categorical variables are expressed as frequencies and percentages. For comparisons between two groups, independent samples t-test was used for normally distributed variables, and the Mann–Whitney U test was applied for non-normally distributed variables. For comparisons among three groups, one-way ANOVA followed by Bonferroni post hoc test was used for normally distributed data, whereas the Kruskal–Wallis test was applied for non-normally distributed data. In the latter case, pairwise comparisons were conducted using the Mann–Whitney U test with Bonferroni correction to adjust for multiple testing. The Chi-square test was used for categorical variables, and Fisher’s exact test was applied when Chi-square assumptions were not met. A p-value < 0.05 was considered statistically significant, with a confidence level of 95%.

## Results

The study included 46 participants with a mean age of 49.4 ± 14.7 years: 16 patients with primary hypophysitis, 9 with NFPA, and 21 controls. Age and sex distribution were comparable across groups (*p* > 0.05). In contrast, when evaluating SUVmax and SUVmean, patients with primary hypophysitis showed higher values than the control group, reaching statistical significance (*p* = 0.018 and *p* = 0.040, respectively); however, although SUVmax and SUVmean values were higher in patients with primary hypophysitis compared with those with NFPA, this difference did not reach statistical significance (*p* > 0.05). Demographic data and SUV values are summarized in Table 1.

**Table 1:** Baseline Demographic Characteristics and SUV Parameters of the Study Groups

	P.H. (*n* = 16)	NFPA (*n* = 9)	Control subjects (*n* = 21)	*p* ^a^	*p* ^b^	*p* ^c^	*p* ^d^
Age, years, mean ± sd	43.3 ± 16.2	54.2 ± 14.5	52.0 ± 12.7	0.115	0.228	0.228	1.000
Gender, F/M, n, (%)	9 (56.2%) / 7 (43.8%)	4 (44%) / 5 (56%)	16 (76.2%) / 5 (23.8%)	0.245	0.688	0.199	0.115
SUVmax, the median [IQR]	4.0 [3.1–7.7]^a^	3.2 [2.1–8.3]	2.9 [2.3–3.6]	**0.023**	0.496	**0.018**	1.000
SUVmean, the median [IQR]	2.9 [2.4–4.0]^b^	2.4 [1.3–4.0]	1.9 [1.7–2.3]	**0.045**	0.515	**0.040**	1.000

**F**: Female, **M**: Male, **NFPA**: Non-functioning pituitary adenomas, **P.H.**: Primary Hypophysitis **SUVmax**: Maximum standardized uptake value, **SUVmean**: Mean standardized uptake value, **sd**: Standard deviation

^a^ All groups

^b^ P.H. vs. NFPA

^c^ P.H. vs. Control subjects

^d^ NFPA vs. Control subjects

## Primary hypophysitis

### Clinical presentation and hormonal profile

Demographic, clinical, and radiological characteristics of patients with primary hypophysitis are presented in Table 2. Among patients with primary hypophysitis, the most frequently reported symptoms were polydipsia (50.0%) and polyuria (50.0%), followed by headache (43.8%), weakness (43.8%), and fatigue (43.8%). Visual disturbances were present in four patients (25.0%). Menstrual irregularities were reported by three female patients (33%), while erectile dysfunction was noted in two male patients (28.6%). One patient (6.2%) remained asymptomatic. Among patients with primary hypophysitis, the most common endocrine disturbances were hypopituitarism, hyperprolactinemia, and AVP deficiency, each affecting nearly half of the cohort. Central hypocortisolism, hypothyroidism, and hypogonadism were also relatively frequent, occurring in about one-third of patients. Growth hormone deficiency was less common, while hypoprolactinemia was observed only in a single case. The complete set of laboratory parameters is summarized in Table 3.

**Table 2:** Demographic, clinical and radiological data in patients with primary hypophysitis

	*n* = 16
Disease duration, months, mean ± sd	61.1 ± 56.5
*Clinical Features*	
Polydipsia, n (%)	8 (% 50)
Polyuria, n (%)	8 (% 50)
Presence of headache, n (%)	7 (% 43.8)
Weakness, n (%)	7 (% 43.8)
Fatigue, n (%)	7 (% 43.8)
Menstrual irregularities (in females), n (%)	3 (% 33)
Erectile dysfunction (in males), n (%)	2 (% 28.6)
Visual complaints, n (%)	4 (% 25.0)
*Endocrinological Findings*	
Hypopituitarism	7 (% 43.8)
AVP deficiency, n (%)	7 (% 43.8)
Hyperprolactinemia, n (%)	7 (% 43.8)
Central hypocortisolism, n (%)	5 (% 31.3)
Central hypothyroidism, n (%)	5 (% 31.3)
Central hypogonadism, n (%)	5 (% 31.3)
Growth hormone deficiency, n (%)	3 (% 18.8)
Hypoprolactinemia, n (%)	1 (% 6.3)
*Treatment and Imaging Findings*	
Increased infundibular thickness on baseline MRI, n (%)	11 (% 68.8)
Homogeneous contrast enhancement on baseline MRI, n (%)	7 (% 43.8)
T1 hyperintensity on baseline MRI, n (%)	6 (% 37.5)
Optic chiasm compression on baseline MRI, n (%)	1 (% 6.3)
Treatment modality (Follow-up / Steroid), n (%)	10 (% 62.5) / 6 (% 37.5)

**sd**: Standard deviation, **M**: Male, **F**: Female

**Table 3:** Laboratory parameters in patients with primary hypophysitis

	*n* = 16
CRP, median [IQR]	0.8 [0.4–3.1]
ESR, median [IQR]	8.0 [5.0–32.5]
Serum β-hCG, median [IQR]	0.1 [0.1–0.2]
Serum AFP, median [IQR]	2.4 [1.7–4.0]
CSF β-hCG, median [IQR]	0.1 [0.1–0.3]
CSF AFP, median [IQR]	0.7 [0.2–1.4]
Serum ACE level, median [IQR]	22.5 [15.0–31.5]
24-hour urinary calcium level, median [IQR]	178.2 [120.2–280.6]
C-ANCA level, median [IQR]	1.0 [0.4–1.7]
P-ANCA level, median [IQR]	0.8 [0.7–4.2]
LDH, median [IQR]	256.0 [181.0–290.0]
IgG4 level, median [IQR]	1.4 [0.3–104.7]
GH level, median [IQR]	0.4 [0.1–1.0]
IGF-1 level, median [IQR]	163.8 [85.7-207.2]
Prolactin level, median [IQR]	24.9 [16.7–34.8]
FSH level, median [IQR]	4.1 [0.9–7.3]
LH level, median [IQR]	3.7 [0.5–6.6]
Estradiol level, median [IQR]	12.8 [5.0–38.3]
Testosterone level, median [IQR]	240.3 [170.6–837.7]
ACTH level, median [IQR]	18.1 [9.1–31.5]
Basal cortisol level, median [IQR]	9.7 [4.8–14.5]
TSH level, median [IQR]	1.5 [0.2-2.0]
Free T4 level, median [IQR]	1.2 [1.0–1.4]

**GH**: Growth hormone, **FSH**: Follicle stimulating hormone, **LH**: luteinizing hormone, **ACTH**: Adrenocorticotropic hormone, **IGF-1**: Insulin-like growth factor-1,

**TSH**: Thyroid stimulating hormone, **CRP**: C-reactive protein, **ESR**: Erythrocyte sedimentation rate, **β-hCG**: β-human chorionic gonadotropin, **AFP**: Alpha-fetoprotein, **CSF**: Cerebrospinal fluid, **ACE**: angiotensin-converting enzyme,

**C-ANCA**: Antineutrophil cytoplasmic antibodies, **P-ANCA**: Perinuclear anti-neutrophil cytoplasmic antibody, **LDH**: Lactate dehydrogenase

### Treatment

Steroid therapy was administered to six patients (37.5%), and ten patients (62.5%) were managed with follow-up only and surgical treatment was not required in any case.

### Correlation analysis

Among patients with primary hypophysitis, no significant correlations were observed between SUVmax and SUVmean values and clinical or laboratory parameters (*p* > 0.005).

### ROC analysis

ROC analysis was performed only between the primary hypophysitis group and the control group without any intracranial pathology.

**Fig. 1:**
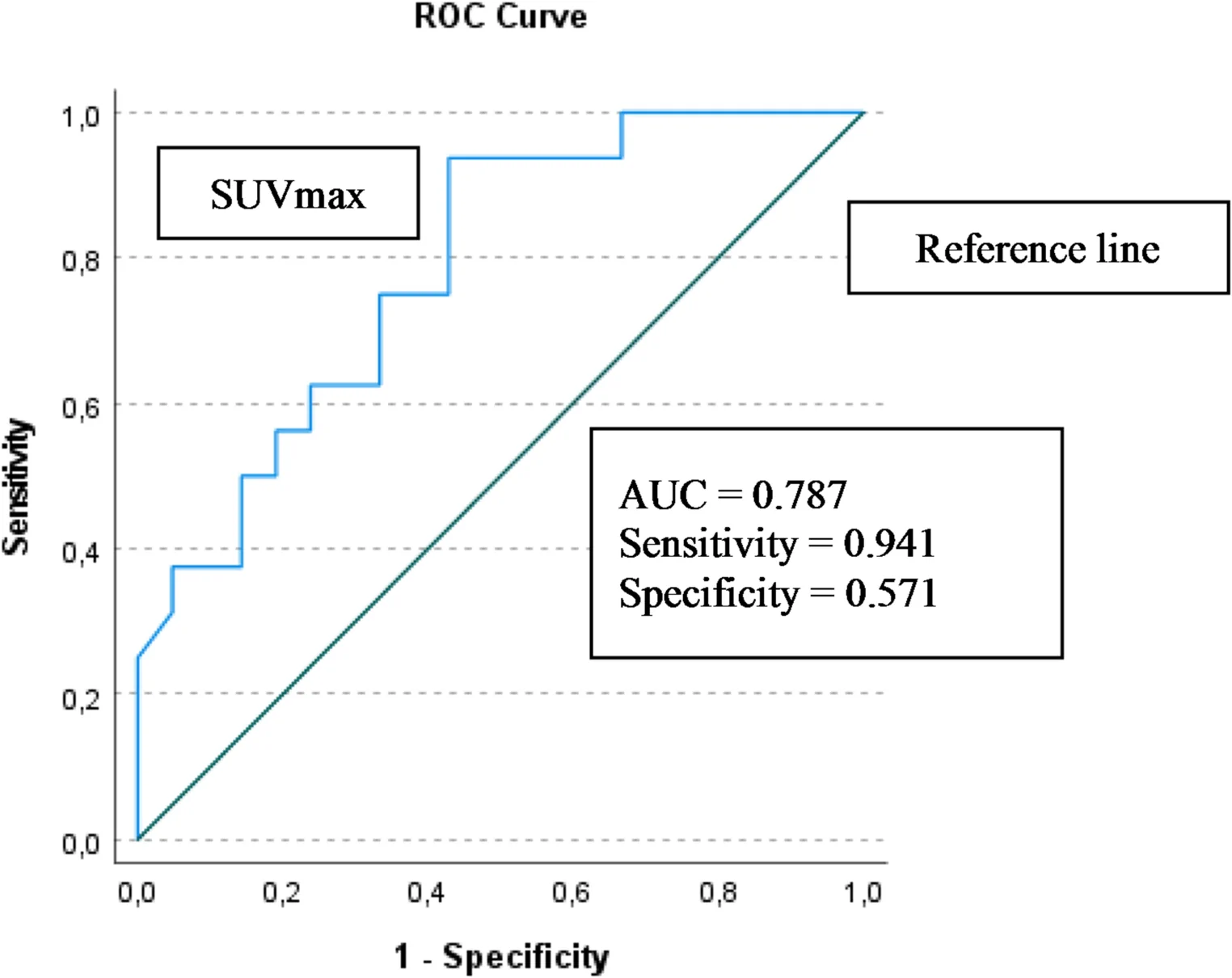
ROC curve analysis of ¹⁸F-FDG PET SUVmax for the prediction of primary hypophysitis compared with controls (16 patients with primary hypophysitis vs. 21 controls without intracranial pathology). AUC = 0.787 (95% CI: 0.643–0.932; *p* < 0.001). The optimal cut-off (SUVmax ≥ 2.98) yielded 94.1% sensitivity and 57.1% specificity

In the ROC analysis of SUVmax at diagnosis, the AUC was 0.787, (95% CI: 0.643–0.932; *p* < 0.001), indicating moderate discriminatory power. The optimal cut-off value was determined as SUVmax ≥ 2.9750, which corresponded to a sensitivity of 94.1%, specificity of 57.1%, and a positive predictive value (PPV) of 64%. The model demonstrated an accuracy rate of 73.7% and an F1 score of 0.76, reflecting a good balance between sensitivity and predictive performance (Fig. 1).

**Fig. 2:**
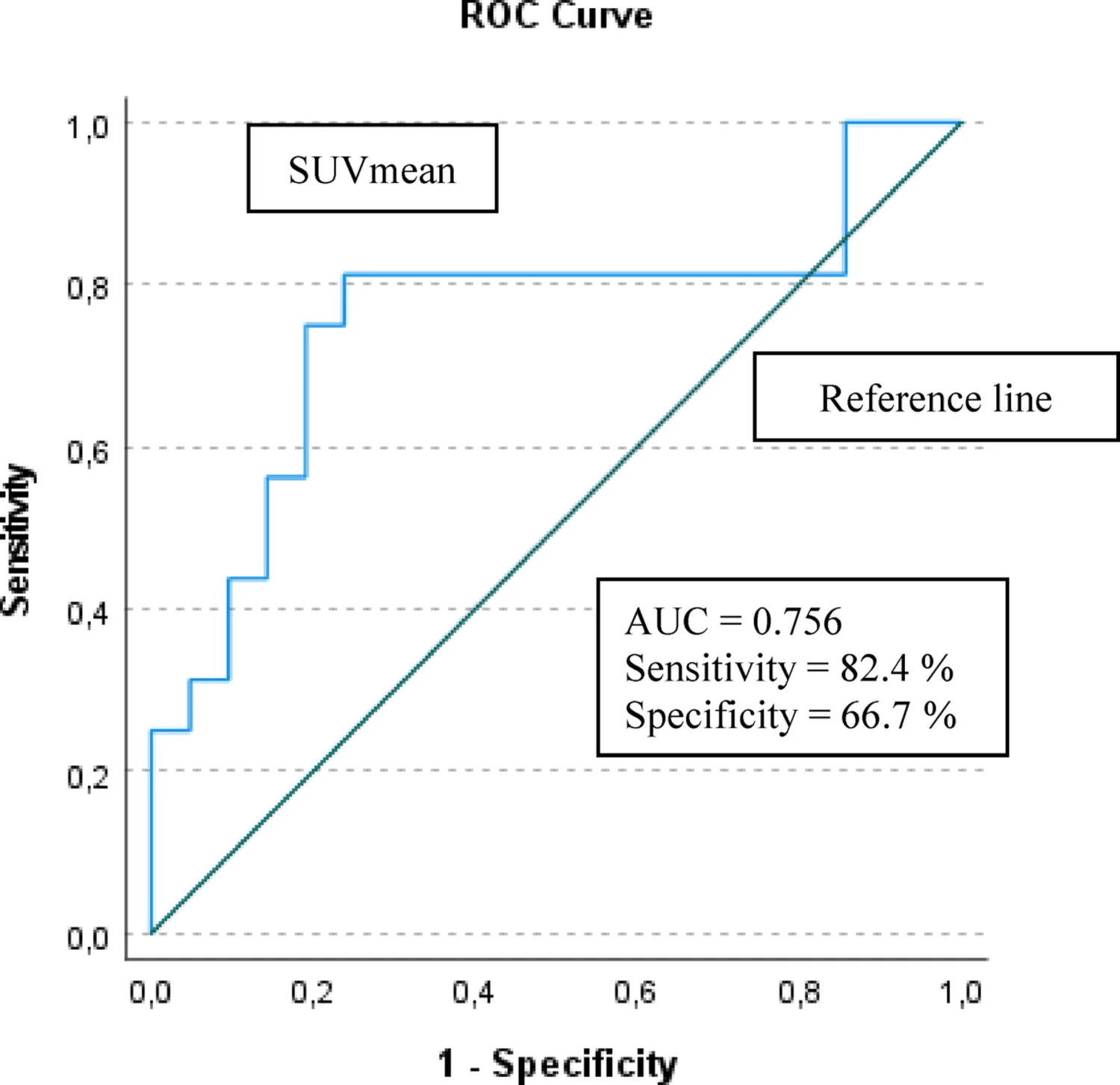
ROC curve analysis of ¹⁸F-FDG PET SUVmean for the prediction of primary hypophysitis compared with controls (16 patients with primary hypophysitis vs. 21 controls without intracranial pathology). AUC = 0.756 (95% CI: 0.583–0.929; *p* = 0.004). The optimal cut-off (SUVmean ≥ 2.20) provided 82.4% sensitivity and 66.7% specificity

In the ROC analysis of SUVmean at diagnosis, the AUC was 0.756 (95% CI: 0.583–0.929; *p* = 0.004), indicating significant discriminatory power. The optimal cut-off value was SUVmean ≥ 2.195, yielding a sensitivity of 82.4%, specificity of 66.7%, and a PPV of 66.7%. The corresponding accuracy rate was 73.7%, with an F1 score of 0.73 (Fig. 2).

### ^18^F-FDG PET imaging during follow-up

**Fig. 3:**
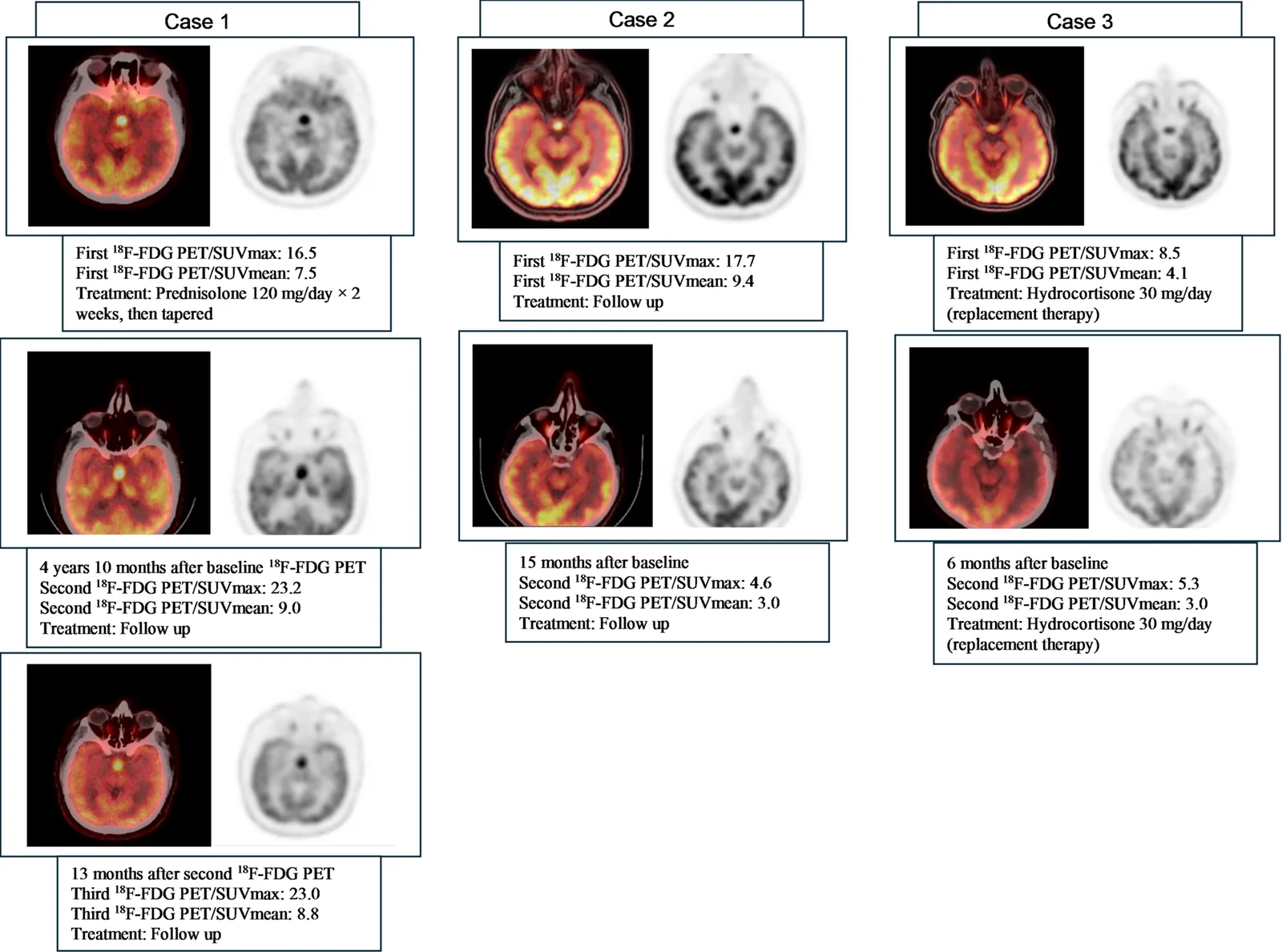
Representative ¹⁸F-FDG PET images of three patients with primary hypophysitis evaluated during follow-up. One patient received high-dose corticosteroid therapy, another received replacement-dose corticosteroids, and the third was managed conservatively without intervention

^18^F-FDG PET imaging was performed during follow-up in three patients with primary hypophysitis. Among them, one received high-dose corticosteroid therapy, another was treated with replacement dose corticosteroid therapy, while the third followed without intervention. Representative ^18^F-FDG PET images along with corresponding SUVmax and SUVmean values are presented in Fig. 3.

### ^18^F-FDG PET uptake according to clinical and laboratory parameters

^18^F-FDG PET uptake did not differ significantly between treatment modalities (follow-up alone vs. steroid therapy). At diagnosis, patients with hypopituitarism showed higher SUV values than those without, with SUVmean reaching statistical significance (*p* = 0.044) and SUVmax displaying a trend toward significance (*p* = 0.061). Similarly, patients with AVP deficiency had increased uptake, with SUVmean significantly elevated (*p* = 0.050), while SUVmax did not reach significance (*p* = 0.099). In the growth hormone deficiency group, both SUVmax and SUVmean were significantly higher compared with patients without this condition (*p* = 0.020 and *p* = 0.043, respectively). On sellar MRI, homogeneous enhancement was associated with markedly lower SUV values compared to non-homogeneous enhancement (*p* = 0.008 and *p* = 0.012). No further associations were detected between ^18^F-FDG PET uptake and clinical, laboratory, or MRI findings (*p* > 0.05; Table 4).

**Table 4:** Comparison of 18 F-FDG PET SUVmax and SUVmean values according to demographic, clinical, and radiological features in patients with primary hypophysitis

	SUVmax	*p* ^a^	SUVmean	*p* ^b^
Male vs. Female	8.1 ± 6.4 / 4.3 ± 1.9	0.178	4.3 ± 3.0 / 3.1 ± 1.2	0.368
*Clinical Features*				
Headache, Presence vs. Absence, mean ± sd	3.8 ± 0.8 / 7.6 ± 5.8	0.090	2.8 ± 0.6 / 4.2 ± 2.7	0.153
Polydipsia, Presence vs. Absence, mean ± sd	7.7 ± 6.1 / 4.2 ± 1.9	0.170	4.5 ± 2.7 / 2.7 ± 0.7	0.112
Polyuria, Presence vs. Absence, mean ± sd	7.7 ± 6.1 / 4.2 ± 1.9	0.170	4.5 ± 2.7 / 2.7 ± 0.7	0.112
Weakness, Presence vs. Absence, mean ± sd	8.1 ± 6.4 / 4.2 ± 1.9	0.166	4.4 ± 2.9 / 3.0 ± 1.3	0.269
Fatigue, Presence vs. Absence, mean ± sd	8.1 ± 6.4 / 3.6 ± 0.5	0.111	4.4 ± 2.9 / 2.6 ± 0.6	0.160
Visual complaints, Presence vs. Absence, mean ± sd	3.9 ± 0.4 / 6.6 ± 5.3	0.104	3.1 ± 0.4 / 3.8 ± 2.5	0.359
*Endocrinological Findings*				
Hypopituitarism, Presence vs. Absence, mean ± sd	7.5 ± 5.7 / 3.6 ± 0.9	0.061	4.5 ± 2.4 / 2.5 ± 0.5	**0.044**
Central hypocortisolism, Presence vs. Absence, mean ± sd	7.1 ± 5.4 / 5.4 ± 4.4	0.579	3.8 ± 2.2 / 3.5 ± 2.2	0.792
Central hypothyroidism, Presence vs. Absence, mean ± sd	8.2 ± 5.4 / 4.9 ± 4.2	0.276	4.4 ± 2.3 / 3.2 ± 2.1	0.346
Central hypogonadism, Presence vs. Absence, mean ± sd	8.3 ± 5.3 / 4.9 ± 4.3	0.258	4.6 ± 2.1 / 3.1 ± 2.1	0.241
Hyperprolactinemia, Presence vs. Absence, mean ± sd	6.3 ± 4.9 / 5.7 ± 4.8	0.795	3.9 ± 2.0 / 3.4 ± 2.3	0.655
Growth hormone deficiency, Presence vs. Absence, mean ± sd	11.5 ± 4.3 / 4.7 ± 3.9	**0.020**	5.9 ± 1.7 / 3.1 ± 1.9	**0.043**
AVP deficiency, Presence vs. Absence, mean ± sd	8.2 ± 6.4 / 4.2 ± 1.7	0.099	4.8 ± 2.8 / 2.7 ± 0.7	0.050
Treatment and Imaging Findings				
Increased infundibular thickness on baseline MRI, Presence vs. Absence, mean ± sd	6.6 ± 5.4 / 4.5 ± 2.7	0.319	3.9 ± 2.3 / 3.0 ± 1.7	0.416
T1 hyperintensity on baseline MRI, Presence vs. Absence, mean ± sd	7.2 ± 5.4 / 5.2 ± 4.4	0.476	4.3 ± 2.5 / 3.2 ± 1.9	0.372
Homogeneous contrast enhancement on baseline MRI, Presence vs. Absence, mean ± sd	3.9 ± 2.4 / 7.4 ± 5.3	**0.008**	2.6 ± 1.5 / 4.3 ± 2.2	**0.012**
Treatment, Follow up / Steroid, mean ± sd	6.2 ± 5.7 / 5.5 ± 2.8	0.746	3.7 ± 2.5 / 3.4 ± 1.5	0.730

sd: Standard deviation, M: Male, F: Female, ^a^p value for SUVmax, ^b^ p value for SUVmean

## Discussion

The present study provides novel insights into the metabolic profile of primary hypophysitis using ^18^F-FDG PET imaging. We found that patients with primary hypophysitis had significantly higher SUVmax and SUVmean values compared with healthy controls, highlighting increased metabolic activity in the disease state. Notably, increased ^18^F-FDG PET uptake was most prominent among patients with hypopituitarism, AVP deficiency, or growth hormone deficiency, and in those with homogeneous sellar enhancement on MRI at diagnosis. These observations point toward a relationship between metabolic activity, disease severity, and radiological presentation.

Publications investigating the relationship between ^18^F-FDG PET and hypophysitis have primarily focused on incidental findings or cases induced by immunotherapy. The first report was a 2013 case study in which ^18^F-FDG PET/CT demonstrated ipilimumab-related hypophysitis in a patient with advanced melanoma, with FDG uptake normalizing after steroid therapy [10]. In a subsequent study, Fischer et al. evaluated 14 patients with immunotherapy-induced hypophysitis and demonstrated significantly higher target-to-background ratios (TBR) compared with controls, achieving 72.7% sensitivity and 90.9% specificity at an optimal threshold [16]. Galligan et al. further investigated 162 melanoma patients treated with immunotherapy, identifying increased pituitary uptake in 26.9% of cases, typically within the first three months of treatment; uptake was transient and resolved within six months [17]. While these findings highlight the utility of ^18^F-FDG PET in immunotherapy-related hypophysitis, data on primary hypophysitis remain limited. In a large retrospective series, Ju et al. reported incidental pituitary FDG uptake in 19 patients, among whom only one case was biopsy-proven primary hypophysitis [8]. In our study, which included 16 patients with primary hypophysitis, SUVmax and SUVmean values were significantly higher than in controls. These results underscore the potential role of ^18^F-FDG PET imaging in the evaluation of primary hypophysitis, although further studies with larger cohorts are warranted to confirm its diagnostic and clinical relevance.

An important challenge in the management of primary hypophysitis is determining the most appropriate treatment approach, whether to proceed with or without intervention. Although several studies have explored potential predictors of treatment response, definitive criteria remain lacking. For example, Chiloiro et al. reported that in patients receiving immunosuppressive therapy, response was associated with the presence of anti-pituitary antibodies, AVP deficiency at diagnosis, absence of the physiological neurohypophyseal bright spot on T1-weighted imaging, and pituitary stalk thickness greater than 3.9 mm [18]. Similarly, Lupi et al. identified AVP deficiency as a negative prognostic factor, likely reflecting irreversible damage from pituitary stalk infiltration [19]. Because the number of patients in our cohort who experienced recurrence or progression was limited, we were unable to directly assess the association of ^18^F-FDG PET uptake with disease outcomes. In our study, patients with primary hypophysitis who developed hypopituitarism exhibited significantly higher SUV uptake compared with patients without endocrine dysfunction. Although it is premature to assert that SUV values can reliably predict treatment response, these findings suggest that ^18^F-FDG PET-derived metabolic parameters may hold promise as prognostic biomarkers, potentially aiding in the determination of optimal timing for medical intervention. Given that immunosuppressive therapy is believed to be more effective during the early and active stages of primary hypophysitis, the observed increase in FDG uptake may potentially reflect inflammatory activity within the pituitary gland. However, due to the limited number of patients with longitudinal follow-up and treatment response data in our study, no definitive conclusions can be drawn regarding the role of ^18^F-FDG PET imaging as a marker of disease activity. Prospective studies with serial ^18^F-FDG PET evaluations are needed to clarify this potential association.

One of the critical challenges in the diagnosis of hypophysitis is its differentiation from NFPAs. The presence of cases misdiagnosed as NFPA and subsequently referred for surgery underscores the clinical importance of this distinction [20, 21]. To aid in this process, various scoring systems and artificial intelligence–based approaches have been proposed [22, 23]. Although ^18^F-FDG PET imaging has been utilized in patients with non-functioning pituitary adenomas, its specific role in differentiating primary hypophysitis has not yet been systematically investigated. In pituitary adenomas, however, increased metabolic activity on ^18^F-FDG PET —particularly in macroadenomas—has been consistently demonstrated. However, comparisons between NFPA and functional pituitary adenomas (FPAs) have produced inconsistent results, with some studies showing higher uptake in NFPA and others reporting comparable levels [24–27]. Our study is the first to evaluate the role of ^18^F-FDG PET in differentiating primary hypophysitis from NFPA. Although SUV values were higher in patients with primary hypophysitis, the difference did not reach statistical significance. This absence of significance may be explained by the biological heterogeneity of the NFPA group. Variations in proliferative activity likely influence glucose metabolism, and the inclusion of both macroadenomas and microadenomas—lesions with distinct metabolic profiles—may have introduced additional variability. These factors may have obscured a potential difference between the groups. Taken together, these findings indicate that ^18^F-FDG PET does not provide a clear distinction between primary hypophysitis and NFPA, likely due to overlapping metabolic activity between the two entities.

Several limitations should be acknowledged when interpreting our findings. First, the overall sample size was relatively small, which may have limited the statistical power of subgroup analyses across all study groups. Therefore, subgroup findings should be interpreted with caution. Second, tissue diagnosis of NFPA was not available for all patients, preventing definitive exclusion of silent pituitary adenomas and restricting evaluation of tumor proliferative potential. Third, the NFPA cohort was heterogeneous, comprising both macroadenomas and microadenomas, which differ in biological behavior and glucose metabolism. These factors may have introduced variability and should be carefully considered when interpreting the study outcomes. In addition, a subset of patients included in the present study had been previously reported in a larger Turkish hypophysitis cohort [28]; however, the current analysis focuses specifically on ^18^F-FDG PET imaging parameters, which were not systematically evaluated in the prior publication.

In conclusion, ¹⁸F-FDG PET imaging may serve as a useful adjunct in the evaluation of primary hypophysitis. In our cohort, patients with primary hypophysitis demonstrated higher metabolic activity compared with controls, particularly among those with hypopituitarism, AVP deficiency, or homogeneous sellar enhancement on MRI—features that may reflect more extensive inflammatory involvement. These findings suggest that ^18^F-FDG PET imaging provides complementary metabolic information beyond conventional radiological and clinical assessment. While ^18^F-FDG PET may potentially offer insights into disease activity and contribute to therapeutic decision-making, the current evidence remains insufficient to establish its definitive diagnostic or prognostic role. Larger, prospective studies incorporating longitudinal ^18^F-FDG PET evaluation are needed to clarify its clinical utility in the management of primary hypophysitis.
